# Multiple rearrangements in cryptic species of electric knifefish, *Gymnotus carapo *(Gymnotidae, Gymnotiformes) revealed by chromosome painting

**DOI:** 10.1186/1471-2156-11-28

**Published:** 2010-04-27

**Authors:** Cleusa Y Nagamachi, Julio C Pieczarka, Susana SR Milhomem, Patricia CM O'Brien, Augusto CP de Souza, Malcolm A Ferguson-Smith

**Affiliations:** 1 Laboratório de Citogenética, Instituto de Ciências Biológicas, Universidade Federal do Pará, Belém, Pará, Brazil; 2 Cambridge Resource Centre for Comparative Genomics, Department of Veterinary Medicine, University of Cambridge, Cambridge, UK; 3 Centro Federal de Educação Tecnológica, Belem, Brazil

## Abstract

**Background:**

*Gymnotus *(Gymnotidae, Gymnotiformes) is the Neotropical electric fish genus with the largest geographic distribution and the largest number of species, 33 of which have been validated. The diploid number varies from 2n = 39-40 to 2n = 54. Recently we studied the karyotype of morphologically indistinguishable samples from five populations of *G. carapo sensu stricto *from the Eastern Amazon of Brazil. We found two cytotypes, 2n = 42 (30 M/SM + 12 ST/A) and 2n = 40 (34 M/SM + 6 ST/A) and we concluded that the differences between the two cryptic species are due to pericentric inversions and one tandem fusion.

**Results:**

In this study we use for the first time, whole chromosome probes prepared by FACS of the *Gymnotus carapo sensu strictu *species, cytotype with 2n = 42. Using two color hybridizations we were able to distinguish pairs 1, 2, 3, 7, 9, 14, 16, 18, 19, 20 and 21. It was not possible to separate by FACS and distinguish each of the following chromosome pairs even with dual color FISH: {4,8}; {10,11}; {5,6,17}; {12,13,15}. The FISH probes were then used in chromosome painting experiments on metaphases of the 2n = 40 cytotype. While some chromosomes show conserved synteny, others are rearranged in different chromosomes. Eight syntenic associations were found.

**Conclusions:**

These results show that the karyotype differences between these cryptic species are greater than assumed by classical cytogenetics. These data reinforce the previous supposition that these two cytotypes are different species, despite the absence of morphological differences. Additionally, the homology of repetitive DNA between the two provides evidence of recent speciation.

## Background

Cross-species FISH using whole chromosome painting is widely used for phylogenomic studies in many vertebrate groups, including primates [1-7], bats [8,9], deer [10], birds [11,12], etc. These studies make important contributions to our understanding of genomic reorganization and mechanisms of chromosome evolution in warm-blooded vertebrates.

Research using chromosome painting in fishes is unusual. It has been used only with probes made by microdissection [13-19] or by CGH, the latter without defining chromosome pairs [20]. The probes made by flow cytometry have higher complexity than those made by microdissection, and are more appropriate for cross-species hybridization [21]. However, there are no reports in the literature of FACS (Fluorescent Activated Chromosome Sorting) generated probes for chromosome painting involving a whole fish genome.

The chromosome structure of a fish, a cold-blooded vertebrate, lacks both GC-rich and GC-poor compartments. The absence of compartmentalization of their genomes may be the reason for failure to obtain good G-bands (for revision, see [22]). Also, this could explain the difficulty in getting whole chromosome-specific probes by FACS. As well, fish chromosomes are small and there is not enough difference in size to allow the separation of each individual pair. An additional difficulty is to obtain fibroblast cultures and chromosome preparations of high quality and with sufficient metaphases.

*Gymnotus *(Gymnotidae - Gymnotiformes) is the Neotropical electric knifefish genus with the largest geographic distribution, occurring from southern Mexico to northern Argentina [23]. It is also the most diverse known Gymnotiformes genus, with 33 validated species, of which 18 are known to occur in the Amazon basin [24,23,26]. Previous cytogenetic studies show that the diploid number in this genus ranges from 2n = 39-40 (with sex chromosomes of the type X_1_X_2_Y) to 2n = 54, exhibiting variation in the karyotype formula, the quantity of heterochromatin, and the position of the Nucleolar Organization Region [27-30].

*Gymnotus carapo *(holotype from Suriname) was described by Linneaus in the first half of the XVIII century (Albert & Crampton, 2003). Currently it is defined as *G. carapo sensu stricto *and is understood as a complex of morphologically similar or cryptic species, with a large distribution area (Albert & Crampton, 2003). Cytogenetic studies of samples identified as *G. carapo *show different karyotypes: 2n = 54 and 2n = 52 from Southern Brazil, 2n = 48 in Amazonas, 2n = 42 in Pará (reviewed [30]). Recently we studied the karyotype of morphologically indistinguishable individuals from five populations of *G. carapo sensu stricto *from the Eastern Amazon of Brazil. We found two cytotypes, 2n = 42 (30 M/SM + 12 ST/A) and 2n = 40 (34 M/SM + 6 ST/A) and we concluded that the differences between these two cryptic species are due to pericentric inversions and one tandem fusion [31].

In this study we were able to use, for the first time, whole chromosome probes prepared by FACS from a fish, *Gymnotus carapo sensu stricto *species, with 2n = 42 cytotype. These probes were used in hybridizations on metaphases of the cytotype with 2n = 40, to determine the differences between the karyotypes of the two cryptic species.

## Methods

We performed chromosome painting analysis on two specimens of *Gymnotus carapo *from two localities in the Eastern Amazon Basin, Pará State, Brazil: Santa Cruz do Arari (00°42'03.2"S,049°,10'42.1"W) on Marajo Island (MCP 40926) and Almeirim (01°31'34.2"S,052°33'37.9"W) (MPEG 13329). These specimens are part of the sample studied by Milhomem *et al. *[30,31].

A primary fibroblast cell line was established from an electric knifefish *Gymnotus carapo sensu stricto*, cytotype 2n = 42, from Santa Cruz do Arari (Marajo Island). Whole chromosome probes were made from this cell line at the Department of Veterinary Medicine, University of Cambridge, UK. The chromosome specific probes were made by degenerate oligonucleotide primed PCR (DOP-PCR) on flow-sorted chromosomes as described previously [32,10]. Briefly, the chromosomes were prepared as described and stained with Hoechst 33258 (2 mg/mL) and Chromomycin A3 (40 mg/mL) in the presence of magnesium sulfate (2.5 mmol/L) for 2 h. Sodium sulfate (25 mmol/L) and sodium citrate (10 mmol/L) were added 15 min prior to flow sorting. Chromosome sorting was performed using a dual-laser cell sorter (FACStar Plus; Becton Dickinson Immuno-Cytometry Systems). About 400 chromosomes were sorted from each peak in the flow karyotypes. Chromosomes were sorted directly into PCR tubes containing 30 μL distilled water. These samples were amplified by DOP-PCR using the primer 6 MW [32]. Primary PCR products were labelled either with biotin-16-dUTP (Boehringer Mannheim), Fluorescein isothiocyanate (FITC)-12-dUTP (Amersham) or Cy3-dUTP, by taking 1 μL of product to a second round of DOP-PCR using the same primer. The biotin probes were detected with avidin-Cy3 or avidin-Cy5.

The metaphase chromosomal preparations from *Gymnotus carapo sensu stricto*, cytotype with 2n = 40 from Almeirim, were made following the methods described by Bertollo *et al. *[33].

In-situ hybridization of painting probes was performed as previously described [10]. Briefly, 14 μL of the hybridization mixture (50% formamide, 1 × SSC, 10% dextran sulfate, 5 mg salmon sperm DNA) and 1 μL of labeled PCR product were denatured at 65°C for 1 min. In-situ hybridization was performed for 48-72 h at 37°C. The hybridization signal was detected as described earlier [10]. After hybridization and washing of the slides, biotinylated chromosome paints were detected with avidin (Vector Laboratories) coupled with Cy3, CY5 or FITC (Amersham). Probes directly linked to fluorochromes were also used, especially for dual or multi-FISH experiments. DAPI was used as a counterstain. In England, FISH digital images were obtained using a cooled CCD camera (Photometrics NU200 series equipped with a Kodak KAF 1400 chip) coupled to a Zeiss Axiophot microscope. The software Smart Capture VP (Digital Scientific) was used for camera control, digital image acquisition and the merging of DAPI and the fluorochrome images of the paints. In Brazil, digital images were captured with a CCD camera AxioCam Mrm coupled to a Zeiss Axiophot microscope, using Axiovision 3.0 Software (Zeiss). The false color attribution was processed using Axiovision and the brightness and contrast corrected with Adobe Photoshop 7.1.

## Results

Flow sorting of *Gymnotus carapo sensu stricto*, cytotype 2n = 42: Characterization and chromosome identification.

**Figure 1:**
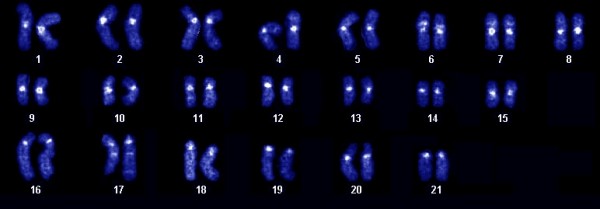
**DAPI Karyotype of *Gymnotus carapo sensu stricto*:** cytotype with 2n = 42 (30 M/SM +12 ST/A).

**Figure 2:**
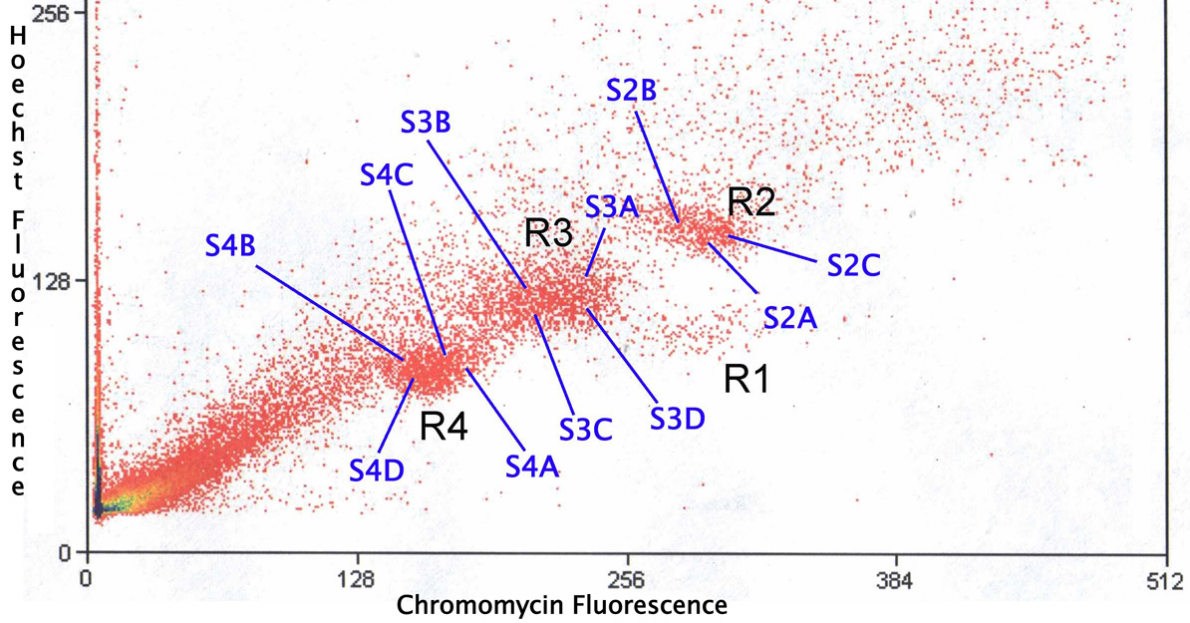
**Bivariate flow karyotype showing 4 Regions: **R1, R2 with 3 subregions (S2A, S2B, S2C), R3 with 4 subregions (S3A, S3B, S3C, S3D) and R4 with 4 subregions (S4A, S4B, S4C, S4D).

**Figure 3:**
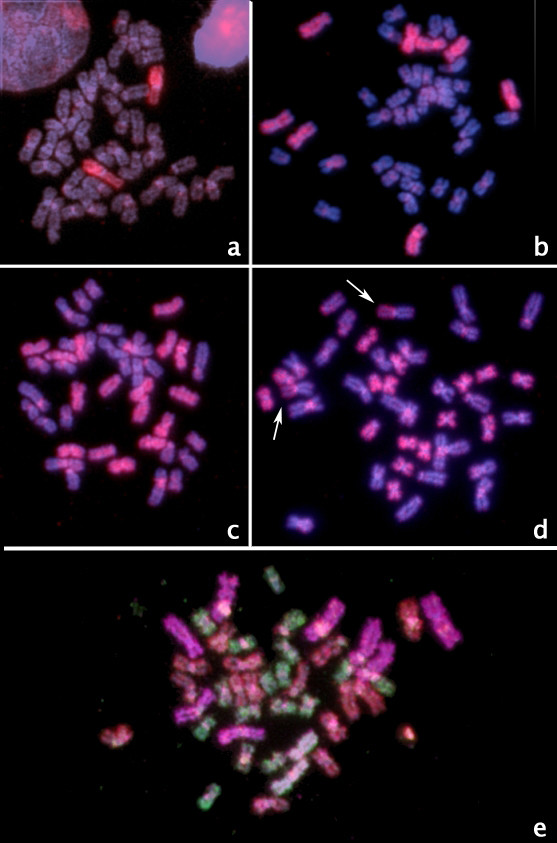
**Hybridization experiments on same species metaphases: a) Region 1 (pair 20, NOR-bearing chromosome); b) Region 2 (pairs 1, 2, 3 and 16); c) Region 3 (pairs 4, 5, 6, 7, 8, 17, 18 and 19); d) Region 4 (pairs 9, 10, 11, 12, 13, 14, 15 and 21); arrows: NORs; e) Multicolor FISH with probes from the four regions: Region 1 = white; Region 2 = purple; Region 3 = red; Region 4 = green**. All the chromosomes were counterstained with DAPI; probes (in red) from "a" to "d", stained with Cy3; 3e) red: Cy3; green: FITC; purple: Cy5; white: a mix of green and purple.

**Figure 4:**
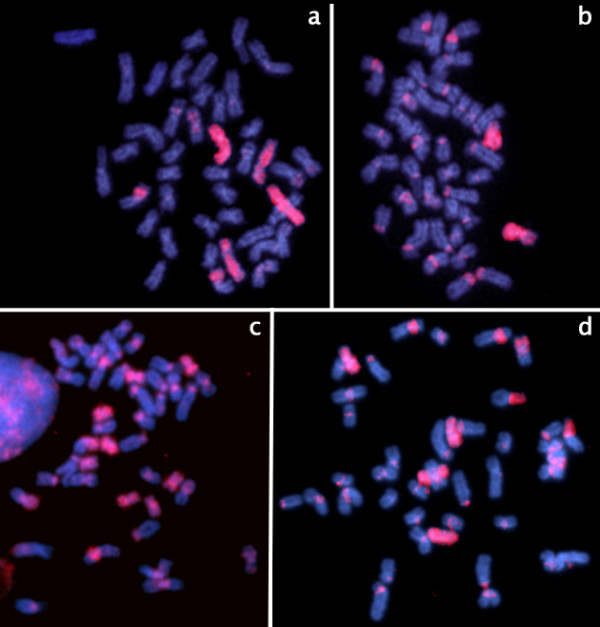
**Examples of same species hybridization with subregions hybridizing fewer chromosomes:** a) S2B (pairs 2 and 16); b) S3C (pair 7); c) S4B (pairs 9, 10, 15 and 21); d) S3B (pairs 7 and 19). All the chromosomes were counterstained with DAPI; probes stained with Cy3.

Figure 1 shows the DAPI banding karyotype of *Gymnotus carapo sensu stricto*, cytotype with 2n = 42 (30 M/SM + 12 ST/A), from which the whole chromosome paints were made. The bivariate flow karyotype shows the four regions (Figure 2), from which whole chromosome probes were produced. Hybridization experiments on metaphases from the same species (Figure 3) show that the only GC-rich region, R1, is represented by the NOR-bearing chromosome (pair 20 - Figure 3a); R2 is represented by the four biggest pairs (1, 2, 3 and 16 - Figure 3b); R3 by the eight medium sized pairs (4, 5, 6, 7, 8, 17, 18 and 19 - Figure 3c) and R4 by the eight smallest pairs (9, 10, 11, 12, 13, 14, 15 and 21 - Figure 3d). A same species multicolor FISH experiment with all the four probes shows that all the 42 chromosomes were hybridized, without superimposition (Figure 3e). From each of the three regions (R2, R3 and R4) further sorting produced three (R2) and four (R3 and R4) subregions (Figure 2). The results of same species hybridization showed that these subregions contain fewer chromosomes (Figure 4). From S2A, S2B, and S2C of R2: S2A contains pairs 1, 2 and 16; S2B contains pairs 2 and 16; S2C contains pairs 1 and 16. From S3A, S3B, S3C, and S3D of R3: S3A contains pairs 5, 6, 7 and 17; S3B contains pairs 7 and 19; S3C contains pair 7; S3D contains pairs 5, 6, 7, 17 and 18. From S4A, S4B, S4C, and S4D of R4: S4A contains pairs 12, 13 and 15; S4B contains pairs 12, 13, 14 and 15; S4C contains pairs 10, 11, 12, 13, 15 and 21; S4D contains pairs 12, 13, 14, 15 and 21. These results are summarized on Table 1. Apart from the homologous sequences, the probes also hybridized to non-specific regions, mainly in centromeric, interstitial, pericentromeric areas and/or the arms of some chromosomes, including the NOR. These areas have highly repetitive DNA.

**Table 1:** Sorted regions of the flow karyotype with corresponding subregions and chromosome numbers.

Region	Number of Chromosomes	Region and Subregion	Number of pairs	Chromosomes
R 1	1	R1	1 pair	20

		R2	4 pairs	1, 2, 3, 16
	
		S2A	3 pairs	1, 2, 16
	
R2	4	S2B	2 pairs	2, 16
	
		S2C	2 pairs	1, 16

		R3	8 pairs	4, 5, 6, 7, 8, 17, 18, 19
	
R 3	8	S3A	4 pairs	5, 6, 7, 17
	
		S3B	2 pairs	7, 19
	
		S3C	1 pair	7
	
		S3D	5 pairs	5, 6, 7, 17, 18

		R4	8 pairs	9, 10, 11, 12, 13, 14, 15, 21
	
R 4	8	S4A	3 pairs	12, 13, 15
	
		S4B	4 pairs	12, 13, 14, 15
	
		S4C	6 pairs	10, 11, 12, 13, 15, 21
	
		S4D	5 pairs	12, 13, 14, 15, 21

Dual color hybridization was used for identification of chromosomes pairs from each of the regions (R2, R3 and R4), as illustrated in Table 2. In brief, it is possible to identify individually pairs 1, 2, 3, 7, 9, 14, 16, 18, 19, 20 and 21, while it was not possible to distinguish each of the following chromosome pairs even with dual color FISH: {4,8}; {10,11}; {5,6,17}; {12,13,15}.

**Table 2:** Dual color FISH used to distinguish the chromosomes pairs of the Regions 2, 3 and 4.

Dual color FISH	Chromosomes pairs
	**Region 2**

S2B × S2C	1 (color of S2C); 2 (color of S2B); 16 (two color)

R2 × S2A	3 (color of R2); 1, 2, 16 (two color)

R2 × S2B	2, 16 (two color); 1, 3 (color of R2) - define pair 3 for exclusion of pair 1

	**Region 3**

S3B × S3C	7 (two color); 19 (color of S3B)

S3A × S3D	18 (color of S3D); 5, 6, 7, 17 (two color)

S3A × S3B	7 (two color); 19 (color of S3B); 5, 6, 17 (color of S3A)

R3 × S3D	5, 6, 7, 17, 18 (two color); 4, 8, 19 (color of R3) - define pairs 4,8 for exclusion of pair 19

	**Region 4**

S4A × S4B	14 (color of S4B); 12, 13, 15 (two color)

R4 × S4C	9, 14 (color of R4); 10, 11, 12, 13, 15, 21 (two color) - define pair 9 for exclusion of pair 14

S4B × S4D	21 (color of S4D); 12, 13, 14, 15 (two color)

S4C × S4D	14 (color of S4D); 10, 11 (color of S4C), 12, 13, 15, 21 (two color)

The dual color FISH allowed the distinction of all four chromosome pairs from Region 2. In Region 3, this allowed the distinction of pairs 7, 18 and 19. It is not possible to distinguish pairs {4, 8} and {5, 6, 17}. In region 4, this allowed the distinction of pairs 9, 14 and 21. It is not possible to distinguish pairs {10, 11} and {12, 13, 15}.

Cross-species hybridization: *Gymnotus carapo sensu stricto*: cytotype 2n = 42 hybridized to cytotype 2n = 40

**Figure 5:**
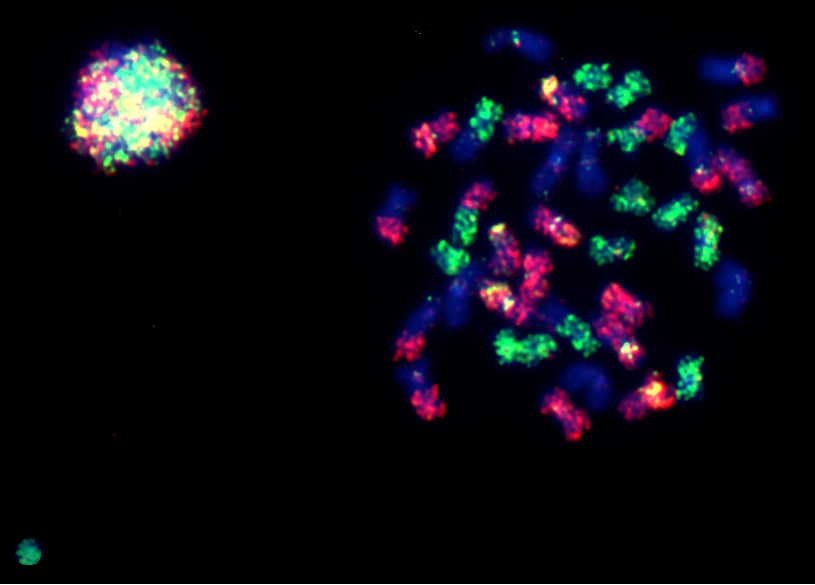
**Cross species hybridization with R3 (red) and R4 (green) for the precise definition of which chromosome or chromosome segment in the 2n = 40 genome corresponds to each chromosome from R3 (pairs 4, 5, 6, 7, 8, 17, 18, 19) and R4 (pairs 9, 10, 11, 12, 13, 14, 15, 21) from cytotype 2n = 42**. All the chromosomes were counterstained with DAPI; red probes: Cy3; green probes: FITC.

**Figure 6:**
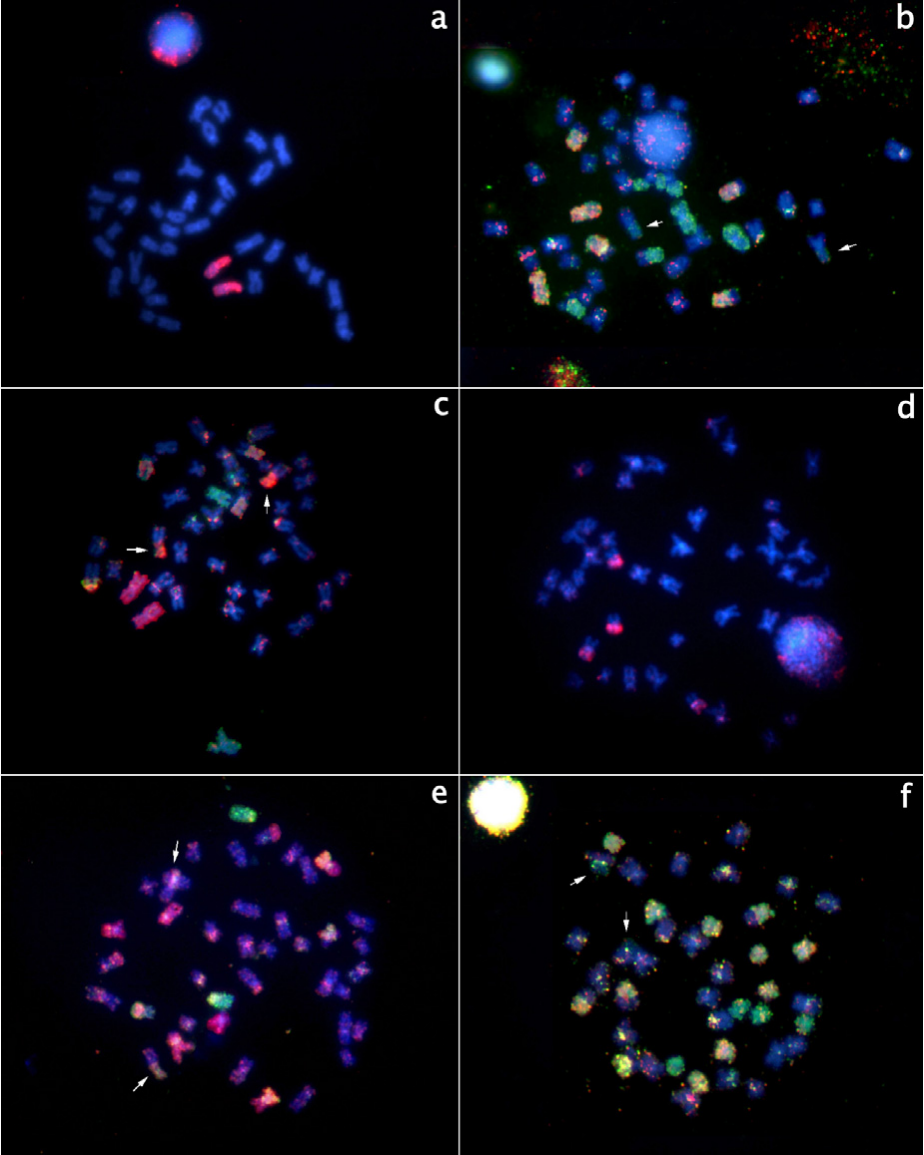
**Examples of cross species hybridizations**. Chromosome numbers refer to the original pairs in *Gymnotus *2n = 42 a) R1 (pair 20 red); b) S2B + R2 (pairs 1 and 3 green; pairs 2 and 16 yellow) c) S2B + S2C (pair 1 red, pair 2 green and pair 16 yellow) d) S3C (pair 7 red); e) S3A + S3B (pair 19 green, pair 7 yellow, pair {5, 6, 17} red); f) S4C + R4 (pairs 9 and 14 green; pairs 10, 11, 12, 13, 15 and 21 yellow). Arrows on b, c, e and f point to the NOR region where nonspecific hybridizations occur. All the chromosomes were counterstained with DAPI; red probes: Cy3; green probes: FITC.

Whole chromosome paints from *Gymnotus carapo sensu stricto*, cytotype 2n = 42, were hybridized on metaphases of the cryptic species with cytotype 2n = 40. For the precise definition of which chromosome belongs to each region, dual colour FISH was used with R3 × R4 (Figure 5). It was possible to define the chromosome or chromosome segment in the 2n = 40 genome that corresponds to each chromosome from R3 (pairs 4, 5, 6, 7, 8, 17, 18, 19) and R4 (pairs 9, 10, 11, 12, 13, 14, 15, 21) from cytotype 2n = 42. The remaining chromosome or chromosome segments correspond to R1 (the easily identified NOR pair - Figure 6a) or R2 (pairs 1, 2, 3, 16 - Figure 6b, c). Using dual color FISH with probes from the same region, it was possible to identify the chromosomes of this region (Figure 6).

**Figure 7:**
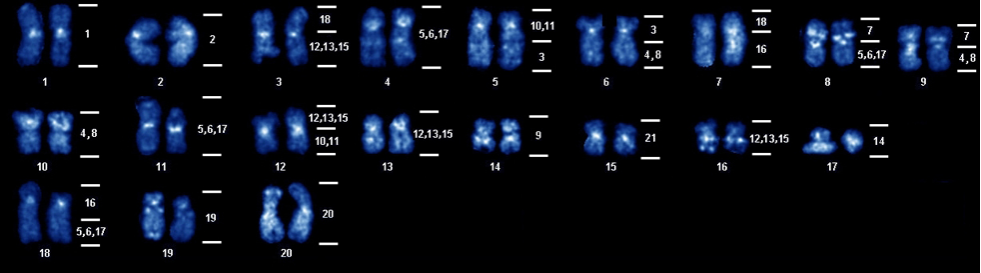
**DAPI karyotype (mounted from the metaphase of Figure 5) of *Gymnotus carapo sensu stricto*, cytotype with 2n = 40 (34 M/SM + 6 ST/A) showing the results of comparative mapping with the chromosomes from the 2n = 42 karyotype (numbers on the right side)**.

Figure 7 shows the karyotype with DAPI banding of *G. carapo *2n = 40 cytotype. Analysis of the hybridizations (Figure 7, Table 3) shows that the pairs 1, 2, 9, 14, 19, 20 and 21 (cytotype 2n = 42) had their synteny conserved and correspond to pairs 1, 2, 14, 17, 19, 20, 15 (cytotype 2n = 40), respectively. Pairs 3, 7, 16 and 18 were rearranged in two parts each. The {10,11} group of chromosomes reveal two separate segments of homology, the {4,8} group reveal three segments and the {5,6,17} and the {12,13,15} groups reveal four segments. The following syntenic associations were found (Figure 7, Table 3): 18/{12,13,15} (pair 3); {10,11}/3 (pair 5); 3/{4,8} (pair 6); 18/16 (pair 7); 7/{5,6,17} (pair 8); 7/{4,8} (pair 9); {12,13,15}/{10,11} (pair 12); 16/{5,6,17} (pair 18).

**Table 3:** Comparative cross species analysis after the definition of groups 3 and 4 (Dual color FISH with R3 and R4 probes).

Region	Pair of 2n = 42	Pair of 2n = 40
R 1	20	20

	1	1
	
R 2	2	2
	
	3	5q distal + 6 (p+q prox.)
	
	16	7q + 18 (p+q prox.)

	{4,8}	6q distal + 9q + 10*
	
R 3	7	8p + 9p
	
	{5,6,17}	4*, 8q, 11*, 18q distal
	
	18	3p + 7p
	
	19	19

	9	14
	
R 4	{10,11}	5p+q prox., 12q
	
	{12,13,15}	3q, 12p, 13, 16
	
	14	17
	
	21	15

* = chromosome pairs with non-specific hybridization on the heterochromatin.

The probes also painted highly repetitive DNA regions in the 2n = 40 genome, usually in a pericentromeric region in a non-specific way. A large region on 4p proximal, 10p proximal and 11q proximal also painted in a non-specific way, probably because of highly repetitive DNA.

## Discussion

*Gymnotus carapo sensu stricto *with 2n = 42 from Marajó Island (Pará-Brazil) is the first fish whose genome was used to generate FACS whole chromosome-specific probes covering the whole karyotype. This is a landmark in the cytogenetic studies of the family Gymnotidae (Gymnotiformes) and the fish in general.

The genome organization of fishes, with its small-sized chromosomes and non-compartmentalization of the chromosomes in GC- or AT-rich sequences (for review, see [22]), makes it difficult to isolate single chromosomes by FACS. As a consequence, most of the probes obtained in our study represent chromosome groups and not single chromosomes. So, for chromosome identification we have to apply a strategy different from that used for warm-blooded vertebrates, in which most probes are actually derived from single chromosomes. Dual color FISH using probes from the same flow karyotype region allowed the identification of many chromosomes individually (pairs 1, 2, 3, 7, 9, 14, 16, 18, 19, 20, 21) except groups: {4,8}, {10,11}, {5,6,17} and {12,13,15}, as shown in Table 2.

The mapping of these probes in the 2n = 40 *G. carapo *cytotype shows surprising and very interesting results. Using classical cytogenetics we supposed that the differences between these karyotypes resulted from a tandem fusion, to explain the diploid number difference, and several pericentric inversions to explain the differences in the karyotypic formula [31]. However, the cross-species FISH made clear that genomic rearrangements were much more frequent than supposed by classical cytogenetics. From the original 21 chromosome pairs of the 2n = 42 *G. carapo *cytotype, only seven chromosomes (pairs 1, 2, 9, 14, 19, 20, 21) have their synteny conserved in the 2n = 40 cytotype. The remaining chromosomes which were individually identified (pairs 3, 7, 16, 18) are all involved in rearrangements with other chromosomes (Figure 7, Table 3). From the chromosomes that remained in groups, the {4,8} group of chromosomes paint a single chromosome (pair 10) and two other chromosomal segments (6q distal and 9q); the {5,6,17} group of chromosomes paint two single chromosomes (pairs 4 and 11) and two segments (8q and 18q distal); the {10,11} group of chromosomes paint two segments (5p+q proximal and 12q); the {12,13,15} group of chromosomes paint chromosomes 13 and 16 and two other chromosomal segments (3q and 12p).

These results show the great importance of comparative genomic mapping using FACS generated whole chromosome-specific probes. In cold-blooded vertebrates like fishes, whose chromosomes do not have a G-banding pattern, the number of rearrangements that differentiate karyotypes is likely to be underestimated. Probably many species with similar 2n and fundamental numbers have many rearrangements, like translocations, that do not change the diploid number and chromosome morphology.

The large genomic reorganization found between the two population cytotypes of *Gymnotus carapo sensu stricto *here studied confirms the hypothesis that they are really different species. The fact that the external morphology, the meristic data and their pigmentation do not allow their distinction [31], suggests that their speciation has been a recent event, in which chromosomal reorganization had a major role.

The paint probes from the 2n = 42 genome also hybridize to highly repetitive DNA regions in the 2n = 40 genome, indicating that this DNA is homologous in both species. Normally, satellite DNA has great sequence divergence between closely related species, despite having great similarity within the repeats of one species [34-36]. The homology of this DNA in both species is additional evidence for recent speciation.

The production of FACS whole chromosome probes from other fishes will be important for understanding chromosomal evolution in vertebrates. Certainly this approach is revealing a wealth of new data in these organisms.

## Conclusion

The whole chromosome probes in *Gymnotus carapo sensu stricto *with 2n = 42 here described and obtained by FACS were used in a cross-species experiment. The chromosome painting demonstrated the large genomic reorganization found between the two cytotypes.

## Authors' contributions

CYN conceived of the study, carried out chromosome painting in *Gymnotus carapo sensu stricto *with 2n = 42 and 2n = 40, organized the data and wrote most of the paper. JCP carried out chromosome painting in *Gymnotus carapo sensu stricto *with 2n = 42 and contributed to the discussion of data. SSRM carried out chromosome painting in *Gymnotus carapo sensu stricto*: cytotype 2n = 40 and contributed to the discussion of data. ACPS collected the samples and helped with the FISH analysis. PCMO'B carried out the whole chromosome probes from *Gymnotus carapo sensu stricto *with 2n = 42 cell lines. MAFS participated of the techniques development and coordinated the study. All authors read and approved the final manuscript.

## References

[B1] Jauch A Wienberg J Stanyon R Arnold N Tofanelli S Ishida T Cremer T Reconstruction of genomic rearrangements in great apes and gibbons by chromosome painting Proceedings of the National Academy of Sciences USA 1992 89 8611 8615 10.1073/pnas.89.18.8611 PMC49970 1528869

[B2] Wienberg J Stanyon R Jauch A Cremer T Homologies in human and *Macaca fuscata *chromosomes revealed by in situ suppression hybridization with human chromosome specific DNA libraries Chromosoma 1992 101 265 270 10.1007/BF00346004 1576879

[B3] Koehler U Bigoni F Wienberg J Stanyon R Genomic reorganization in the concolor gibbon (*Hylobates concolor*) revealed by chromosome painting Genomics 1995 30 287 292 10.1006/geno.1995.9875 8586429

[B4] Neusser M Stanyon R Bigoni F Wienberg J Müller S Molecular cytotaxonomy of New World Monkeys (Platyhrrini). Comparative analysis of five species by multicolor chromosome painting gives evidence for a classification of *Callimico goeldii *within the family of Callitrichidae Cytogenetics and Cell Genetics 2001 94 206 215 10.1159/000048818 11856883

[B5] Barros RMS Nagamachi CY Pieczarka JC Rodrigues LRR Neusser M De Oliveira EHC Wienberg J Muniz JAPC Rissino JD Muller S Chromosomal studies in *Callicebus donacophilus pallescens*, with classic and molecular cytogenetic approaches: Multicolour FISH using human and *Saguinus oedipus *painting probes Chromosome Research 2003 11 327 334 10.1023/A:1024039907101 12906129

[B6] De Oliveira EHC Neusser M Pieczarka JC Nagamachi CY Sbalqueiro IJ Muller S Phylogenetic inferences of Atelinae (Platyrrhini) based on multi-directional chromosome painting in *Brachyteles arachnoides*, *Ateles paniscus paniscus *and *Ateles b. marginatus* Cytogenetic and Genome Research 2005 108 183 190 10.1159/000080814 15545728

[B7] Amaral PJS Finotelo LFM De Oliveira EHC Pissinatti A Nagamachi CY Pieczarka JC Phylogenetic studies of the genus *Cebus *(Cebidae-Primates) using chromosome painting and G-banding BMC Evolutionary Biology 2008 8 169 10.1186/1471-2148-8-169 18534011 PMC2435554

[B8] Pieczarka JC Nagamachi CY O'Brien PCM Yang F Rens W Barros RMS Noronha RCR Rissino J De Oliveira EHC Ferguson-Smith MA Reciprocal chromosome painting between two South American bats: *Carollia brevicauda *and *Phyllostomus hastatus *(Phyllostomidae, Chiroptera) Chromosome Research 2005 13 339 347 10.1007/s10577-005-2886-0 15973499

[B9] Ao L Gu X Feng Q Wang J O'Brien PCM Fu B Mao X Su W Wang Y Volleth M Yang F Nie W Karyotype relationships of six bat species (Chiroptera, Vespertilionidae) from China revealed by chromosome painting and G-banding comparison Cytogenetic and Genome Research 2006 115 145 153 10.1159/000095235 17065796

[B10] Yang F Carter NP Shi L Ferguson-Smith MA A comparative study of karyotypes of muntjacs by chromosome painting Chromosoma 1995 103 642 652 10.1007/BF00357691 7587587

[B11] Guttenbach M Nanda I Feichtinger W Masabanda JS Griffin DK Schmid M Comparative chromosome painting of chicken autosomal paints 1-9 in nine different bird species Cytogenetic and Genome Research 2003 103 173 184 10.1159/000076309 15004483

[B12] De Oliveira EHC de Moura SP dos Anjos LJS Nagamachi CY Pieczarka JC O'Brien PCM Ferguson-Smith MA Comparative chromosome painting between chicken and spectacled owl (*Pulsatrix perspicillata*): implications for chromosomal evolution in the Strigidae (Aves, Strigiformes) Cytogenetic and Genome Research 2008 122 157 162 10.1159/000163093 19096211

[B13] Reed KM Bohlander SK Phillips RB Microdissection of the Y chromosome and fluorescence in situ hybridization analysis of the sex chromosomes of lake trout, *Salvelinus namaycush* Chromosome Research 1995 3 221 226 10.1007/BF00713046 7606359

[B14] Campos-Ramos R Harvey SC Masabanda JS Carrasco LA Griffin DK Identification of putative sex chromosomes in the blue tilapia, *Oreochromis aureus*, through synaptonemal complex and FISH analysis Genetica 2001 111 143 153 10.1023/A:1013707818534 11841163

[B15] Phillips RB Konkol NR Reed KM Stein JD Chromosome painting supports lack of homology among sex chromosomes in *Oncorhynchus*, *Salmo*, and *Salvelinus *(Salmonidae) Genetica 2001 111 119 123 10.1023/A:1013743431738 11841160

[B16] Harvey SC Masabanda J Carrasco LA Bromage NR Penman DJ Griffin DK Molecular-cytogenetic analysis reveals sequence differences between the sex chromosomes of *Oreochromis niloticus*: evidence for an early stage of sex-chromosome differentiation Cytogenetic and Genome Research 2002 97 1-2 76 80 10.1159/000064036 12438743

[B17] Liu JD Yi MS Zhao G Zhou F Wang DQ Yu QX Sex chromosomes in the spiny eel (*Mastacembelus aculeatus*) revealed by mitotic and meiotic analysis Cytogenetic and Genome Research 2002 98 291 297 10.1159/000071051 12826756

[B18] Henning F Trifonov V Ferguson-Smith MA de Almeida-Toledo LF Non-homologous sex chromosomes in two species of the genus *Eigenmannia *(Teleostei: Gymnotiformes) Cytogenetic and Genome Research 2008 121 55 58 10.1159/000124382 18544927

[B19] Henning F Trifonov V de Almeida-Toledo LF Use of chromosome microdissection in fish molecular cytogenetics Genetics and Molecular Biology 2008 31 1 suppl 279 283

[B20] Traut W Winking H Meiotic chromosomes and stages of sex chromosome evolution in fish: zebrafish, platyfish and guppy Chromosome Research 2001 9 8 659 672 10.1023/A:1012956324417 11778689

[B21] Ferguson-Smith MA Yang F O'Brien PCM Comparative mapping using chromosome sorting and painting ILAR Journal 1998 39 2-3 68 76 11528066 10.1093/ilar.39.2-3.68

[B22] Sharma OP Tripathi NK Sharma KK Sobti RC, Obe G, Athwal RS A review of chromosome banding in fishes Some aspects of chromosome structure and function 2002 Narosa Publishing House, New Delhi, India 109 122

[B23] Albert JS Crampton WGR Thorsen DH Lovejoy NR Phylogenetic systematics and historical biogeography of the Neotropical electric fish *Gymnotus *(Teleostei: Gymnotidae) Systematics and Biodiversity 2005 2 4 375 417 10.1017/S1477200004001574

[B24] Albert JS Crampton WGR Five new species of *Gymnotus *(Teleostei: Gymnotiformes) from an Upper Amazon floodplain, with descriptions of electric organ discharges and ecology Ichthyological Exploration of Freshwaters 2001 12 3 241 266

[B25] Albert JS Crampton WGR Seven new species of the Neotropical electric fish *Gymnotus *(Teleostei, Gymnotiformes) with a redescription of *G. carapo *(Linnaeus) Zootaxa 2003 28 1 54

[B26] Crampton WGR Thorsen DH Albert JS Three new species from a diverse, sympatric assemblage of the electric fish *Gymnotus *(Gymnotiformes: Gymnotidae) in the lowland Amazon Basin, with notes on ecology Copeia 2005 1 82 99 10.1643/CI-03-242R2

[B27] Foresti F Almeida-Toledo LF Toledo-Filho SA Chromosome studies in *Gymnotus carapo *and *Gymnotus *sp. (Pisces, Gymnotidae) Caryologia 1984 37 141 146

[B28] Sánchez S Laudicina A Jorge LC A new report of multiple sex chromosome system in the Order Gymnotiformes (Pisces) Cytologia 2004 69 155 160 10.1508/cytologia.69.155

[B29] Silva EB Margarido VP An X1X1X2X2/X1X2Y multiple sex chromosome system in a new species of the genus *Gymnotus *(Pisces, Gymnotiformes) Environmental Biology of Fishes 2005 73 293 297 10.1007/s10641-005-2144-5

[B30] Milhomem SSR Pieczarka JC Crampton WGR de Souza ACP Carvalho JR Nagamachi CY Differences in karyotype between two sympatric species of *Gymnotus *(Gymnotiformes: Gymnotidae) from the Eastern Amazon of Brazil Zootaxa 2007 1397 55 62

[B31] Milhomem SSR Pieczarka JC Crampton WGR Silva DS De Souza ACP Carvalho JR Jr Nagamachi CY Chromosomal evidence for a putative cryptic species in the *Gymnotus carapo *species-complex (Gymnotiformes, Gymnotidae) BMC Genetics 2008 9 75 10.1186/1471-2156-9-75 19025667 PMC2654040

[B32] Telenius H Pelmear A Tunnacliffe A Cytogenetic analysis by chromosome painting using DOP-PCR amplified flow-sorted chromosomes Genes Chromosomes Câncer 1992 4 257 263 10.1002/gcc.2870040311 1382568

[B33] Bertollo LAC Takashi CS Moreira-Filho O Cytotaxonomic considerations on *Hoplias lacerdae *(Pisces, Erythrinidae) Brazilian Journal of Genetics 1978 1 2 103 120

[B34] Dover GA Molecular drive: a cohesive mode of species evolution Nature 1982 299 111 117 10.1038/299111a0 7110332

[B35] Grenier E Castagnone-Sereno P Abad P Satellite DNA sequences as taxonomic markers in nematodes of agronomic interest Parasitology Today 1997 13 10 398 401 10.1016/S0169-4758(97)01113-7 15275154

[B36] Abadon M Grenier E Laumond C Abad P A species-specific satellite DNA from the entomopathogenic nématode *Heterorhabditis indicus* Genome 1998 41 148 153 10.1139/gen-41-2-148 9644825

